# Parecoxib prevents early postoperative cognitive dysfunction in elderly patients undergoing total knee arthroplasty: A double-blind, randomized clinical consort study

**DOI:** 10.1097/MD.0000000000004082

**Published:** 2016-07-18

**Authors:** Yang-Zi Zhu, Rui Yao, Zhe Zhang, Hui Xu, Li-Wei Wang

**Affiliations:** a Department of Anesthesiology, Xuzhou Central Hospital; b Jiangsu Province Key Laboratory of Anesthesiology, Xuzhou Medical University; c Department of neurology, Xuzhou Central Hospital, Xuzhou, PR China.

**Keywords:** inflammation, parecoxib, postoperative cognitive dysfunction, postoperative pain

## Abstract

**Background:**

Trial design neuroinflammation and postoperative pain after surgery are increasingly reported in association with postoperative cognitive dysfunction (POCD). Parecoxib, a selective cyclooxygenase (COX)-2 inhibitor, is used for postoperative analgesia for its potent anti-inflammatory and analgesic effects. This study aimed to evaluate parecoxib's effects on POCD in elderly patients undergoing total knee arthroplasty.

**Methods:**

Around 134 elderly patients undergoing total knee arthroplasty were randomly divided into parecoxib (group P) and control (group C) groups, and treated with parecoxib sodium and saline, respectively, shortly after induction of general anesthesia and 12-h postsurgery, respectively. Perioperative plasma IL-1β, IL-6, TNF-α, and C-reactive protein (CRP) 1evels were measured. Postoperative pain was assessed following surgery. Neuropsychological tests were performed before surgery, and 1 week and 3 months postoperation.

**Results:**

POCD incidence in group P was significantly lower compared with that of group C at 1 week after surgery (16.7% vs 33.9%; *P* < 0.05); no significant difference was found between groups C and P at 3-month follow-up (9.7% vs 6.7%). Compared with group C values, visual analog pain scale (VAS) scores at 3, 6, and 12 hours after surgery were significantly lower in group P(*P* < 0.05). Plasma IL-1β, IL-6, and TNF-α levels were lower in group P than in group C after the operation (*P* < 0.05). No significant difference in the plasma CRP level was found between groups P and C.

**Conclusions:**

Parecoxib sodium decreases POCD incidence after total knee arthroplasty in elderly patients and may explain how this drug suppresses inflammation and acute postoperative pain caused by surgical trauma.

## 1 Introduction

Postoperative cognitive dysfunction (POCD) is a common complication after major surgery in the elderly, and associated with prolonged hospitalization, inability to cope independently, premature unemployment, and possible permanent dementia.^[1–3]^ Although POCD mechanisms remain elusive, mounting evidence has revealed that inflammation plays a key role in the disease process.^[2–4]^ Peripheral inflammation due to surgical trauma and the accompanying release of systemic inflammatory mediators have been shown to influence inflammatory processes in the central nervous system.^[5–8]^ Animal studies indicated that proinflammatory cytokines, such as interleukin 1β (IL-1β) and tumor necrosis factor-α (TNF-a), play a pivotal role in mediating surgery-induced neuroinflammation.^[6,7]^ Indeed, increased expression of proinflammatory cytokines results in performance deficits of hippocampus-dependent cognitive memory.^[5]^ It is well known that inflammation triggered by surgical trauma is also implicated in postoperative pain. Recent studies showed postoperative pain contributes to cognitive dysfunction.^[9,10]^

Selective cyclooxygenase (COX)-2 inhibitor drugs are increasingly used as analgesics in postoperative analgesia. Such drugs attenuate inflammation by decreasing prostaglandin formation via inhibition of COX-2 activity in both peripheral and central tissues. Given their anti-inflammatory and analgesic activities, COX-2 inhibitors might potentially prevent the development of POCD. Animal studies have suggested COX-2 inhibitors to be promising candidates for the treatment of neuroinflammation and cognitive decline caused by surgical trauma.^[11–12]^ However, whether administration of COX-2 inhibitors in the perioperative period can decrease POCD incidence in clinical practice remains unclear.

Therefore, we assessed the effect of the COX-2 inhibitor parecoxib on POCD incidence in aged patients following total knee arthroplasty with general anesthesia; in addition, parecoxib's effects on proinflammatory cytokine expression and visual analog pain scale (VAS) scores in these patients were investigated.

## 2 Methods

### 2.1 Patients and allocation

This was a double-blind, randomized clinical consort study. It was approved by the Ethics Committee of Xuzhou Central Hospital. Written informed consent was obtained from all patients. The sample size of the study was calculated using a free software (http://www.statpages.org) based on a pilot study. Eligible subjects were ASA I or II patients between 65 and 80 years of age, scheduled for total knee arthroplasty. Exclusion criteria were: ASA>II; peptic ulcer disease, cardiac-cerebral vascular disease, diabetes mellitus; neurological or psychiatric disorders; history of allergic reactions to NSAIDs; history of drug and alcohol abuse; hepatic and/or kidney dysfunction; BMI>35; patients on analgesics or antidepressants; Mini-Mental State Examination (MMSE) score < 23; inability to comply with the study protocol or procedures. Using computer-generated randomized table, patients meeting the eligibility criteria were randomized to receive an intravenous bolus of parecoxib sodium 40 mg or placebo (normal saline), shortly after induction of general anesthesia and 12 hours postsurgery, respectively. Patients, anesthetists, and investigators were blinded for treatment allocation.

### 2.2 Anaesthesia and postoperative treatment

All participants received general anesthesia according to a standardized protocol. Anesthesia was induced with 0.1 mg/kg midazolam, 0.2 mg/kg cisatracurium, 2 mg/kg propofol, and 0.6 μg/kg sufentanil, and maintained with remifentanil and propofol. Bispectral Index Score was maintained at 40–60 by adjusting the propofol infusion rate. Heart rate, arterial pressure, respiratory rate, PETCO_2_ and SpO2 were recorded continuously. Patients revived spontaneously without administration of any anesthetic antagonists. Fentanyl was used for patient-controlled analgesia (PCA) and tropisetron was administered for nausea treatment after surgery. Postoperative pain was assessed with a 0 to 10 cm linear visual analog scale (VAS) at 1, 3, 6, 12, 24, and 48 hours after surgery.

### 2.3 Neuropsychological tests

Neuropsychological tests were administered before surgery, and at 1 week and 3 months after surgery. An experienced neurologist, blinded to treatment group assignments, carried out the neuropsychological tests at both times in tranquil surroundings. The test battery, which included 7 tests with 9 subscales, was designed to measure memory, attention and concentration, and psychomotor skills. The tests included: the Mental Control and Digit Span (forward and backward) subtests of the Wechsler Memory Scale (attention and concentration), Visual Retention and Paired Associate Verbal Learning subtests of the Wechsler Memory Scale (figural memory and verbal learning/memory), Digit Symbol subtest of the Wechsler Adult Intelligence Scale-Revised (psychomotor speed), Halstead-Reitan Trail Making Test (Part A) (hand–eye coordination, attention, and concentration), and Grooved Pegboard Test (favored and unfavored hand) (manual dexterity). A decline of 1 or more SDs in 2 or more tests was considered to reflect POCD.^[13]^

### 2.4 Plasma samples collection and detection

Blood samples were collected immediately before induction of anesthesia (T0), and 3 hours (T1), 12 hours (T2), and 48 hours (T3) after surgery. After centrifugation at 3500 g for 10 minutes, plasma samples were stored at −80°C until use. Plasma IL-1β, IL-6, TNF-α, and C-reactive protein (CRP) levels were measured by enzyme-linked immunosorbent assays (ELISAs) following the manufacturer's instructions, with specific kits provided by Dizhao Biologic Project Company, Nanjing, China.

### 2.5 Statistical analysis

Statistical analysis was performed using the SPSS 16.0 software for Windows (SPSS, Chicago, IL). All data were normally distributed as assessed by the Shapiro–Wilk test. Qualitative variables were statistically analyzed with chi-square or Fisher's exact test. Scores of neuropsychological tests between 2 groups were compared by repeated measures ANOVA. Intervention state was the between-subject factor test, whereas time of evaluation was considered the within-subject factor. Independent-samples *t* test or Mann–Whitney *U* test was used for other quantitative variables.

## 3 Results

### 3.1 Patient characteristic

**Figure 1:**
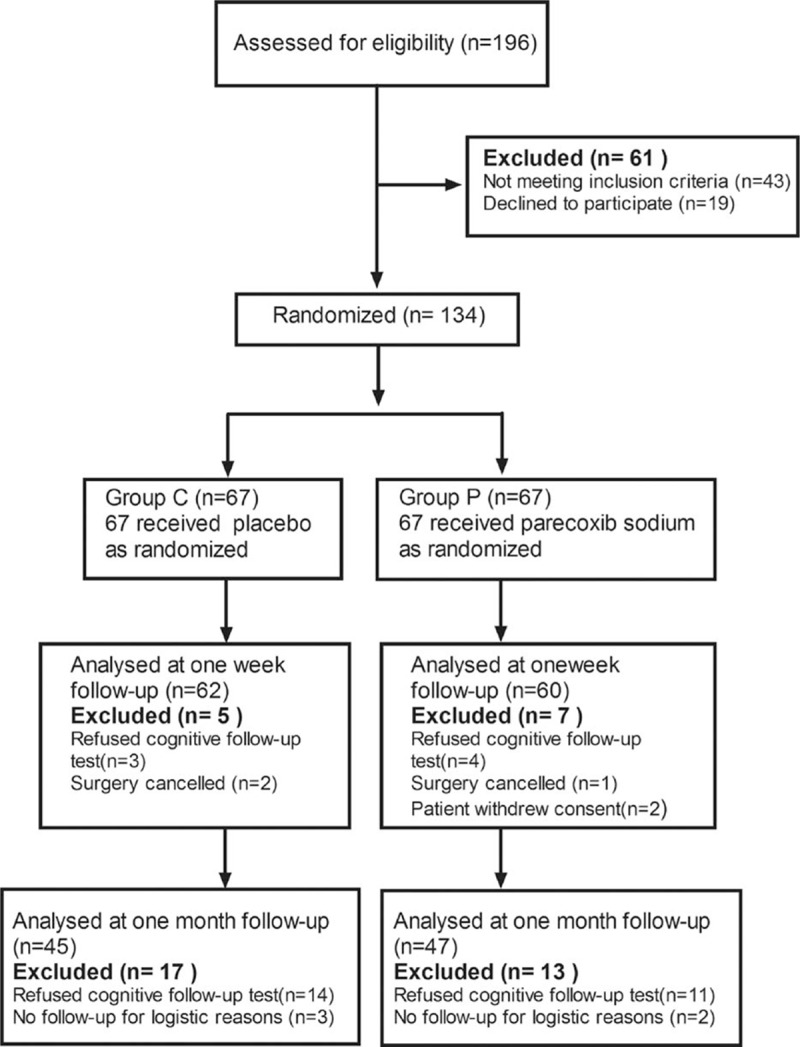
Consort diagram of patients’ randomization, intervention, and analysis.

From February 2014 to May 2015, 134 patients were included in the trial. The flow of patients through the study and detailed reasons for exclusion are provided in Fig. 1. A total of 5 and 7 patients were lost to follow-up at 1-week follow-up in groups C (control) and P (parecoxib treatment), respectively. In addition, 17 and 13 patients were lost to follow-up at 3-month follow-up in control and parecoxib groups, respectively. The basic demographic and clinical characteristics of patients in both groups are presented in Table 1. A significant difference was obtained in smoker number between both groups (*P* < 0.05), but the other characteristics were similar.

**Table 1:**
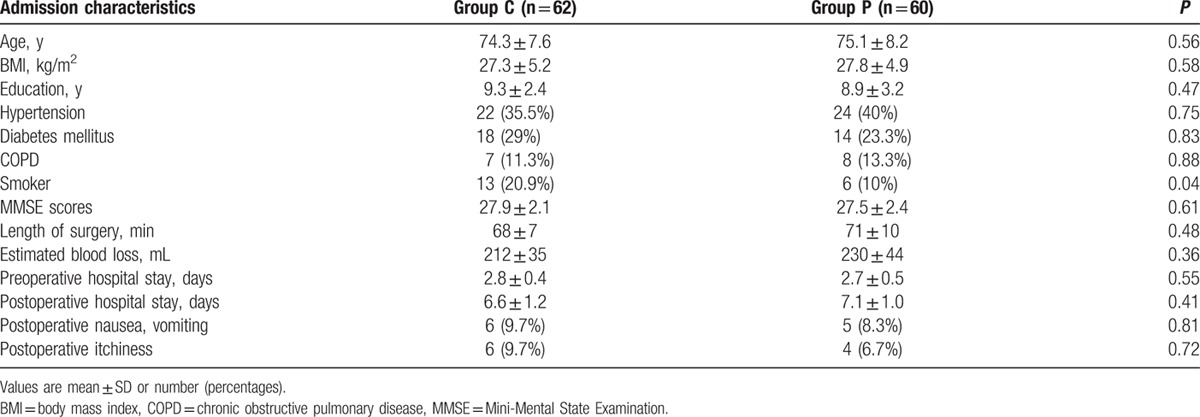
Demographic and clinical characteristics.

### 3.2 Neuropsychological test results and incidence of POCD

There were significant differences in values obtained for mental control, Digit symbol, and Pegboard favored hand between both groups. The control group showed a statistically significant downward trend in values obtained for most neuropsychological tests compared with the parecoxib group (Table 2). Importantly, the parecoxib group showed lower POCD incidence compared with controls at 1-week follow-up (16.7% vs 33.9%, *P* < 0.05). However, no statistically significant difference in POCD incidence between both groups was observed at 3-month follow-up (Table 3).

**Table 2:**
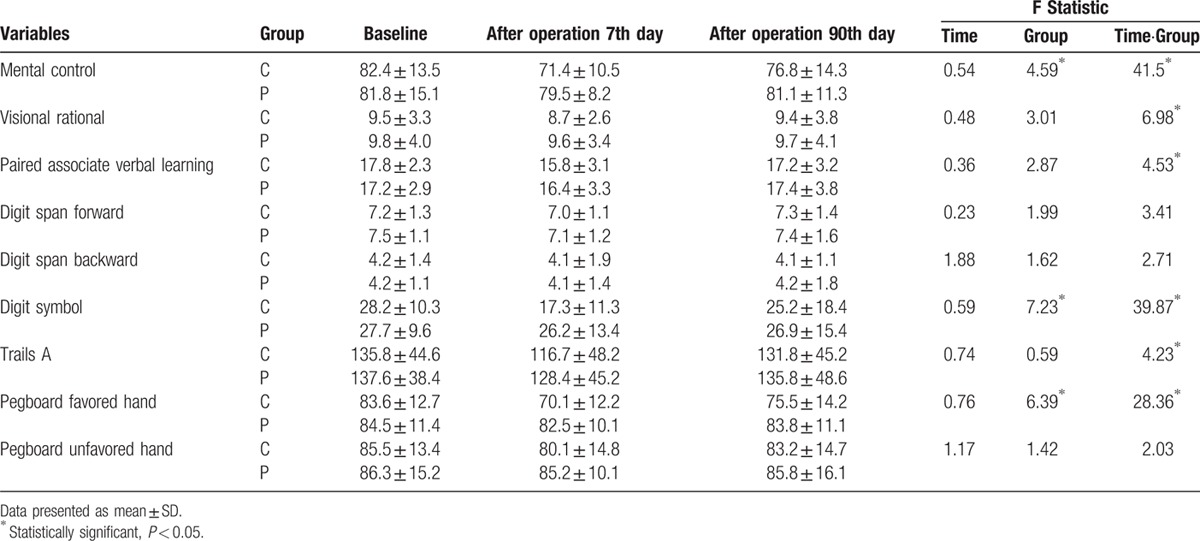
Neuropsychological test results at baseline, 7 days, and 90 days follow-up in patients.

**Table 3:**
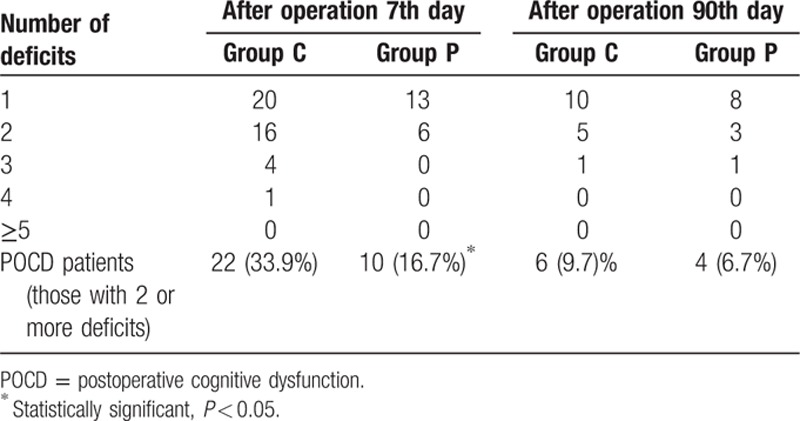
Patients with postoperative neuropsychological deficit in the test battery at baseline, 7 days, and 90 days follow-up.

### 3.3 Postoperative pain and plasma levels of pro-inflammatory cytokines

**Figure 2:**
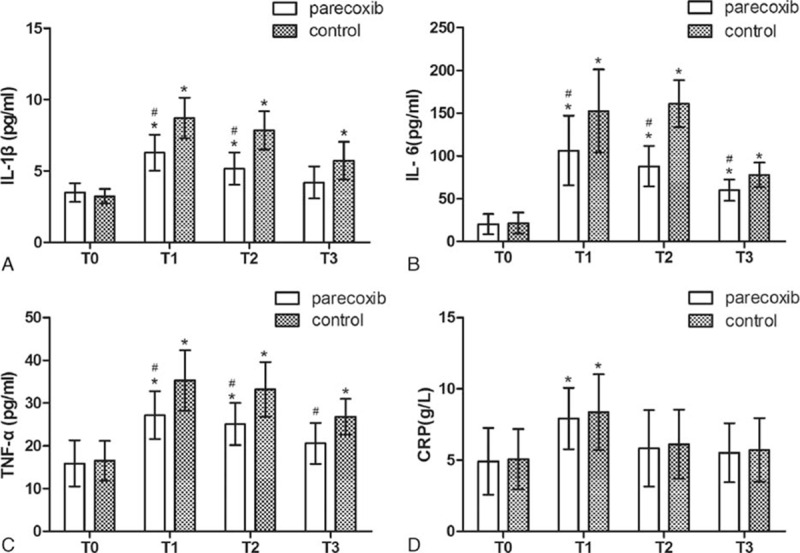
Plasma levels of IL-1β, IL-6, TNF-α, and CRP before and after surgery in parecoxib and control group. ∗*P* < 0.05 versus baseline (T0), ^#^*P* < 0.05 versus control group. CRP = C-reactive protein.

Significantly lower VAS scores were found at 3, 6, and 12 hours in group P, compared to group C values (Table 4). ELISA data showed that plasma levels of IL-1β, IL-6, TNF-α, and CRP were higher after surgery compared with baseline levels in both groups (*P* < 0.05). Group P patients had significantly lower plasma IL-1β, IL-6, and TNF-α levels after surgery compared with group C individuals (*P* < 0.05, Fig. 2A–C). There was no significant difference in plasma CRP levels after surgery between both groups (Fig. 2D).

**Table 4:**
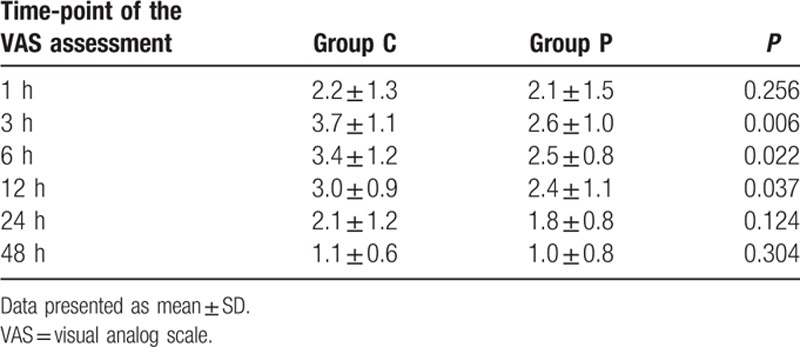
Postoperative pain.

## 4 Discussion

This randomized clinical trial assessing 122 aged patients demonstrated the benefit of parecoxib sodium in decreasing POCD incidence at 1 week postsurgery. In patients treated with parecoxib sodium, serum IL-1β, IL-6, and TNF-α levels were decreased in comparison with control values. Besides, parecoxib sodium decreased postoperative pain scores in patients after surgery. To the best of our knowledge, this is the first randomized clinical trial demonstrating the protective effect of parecoxib on POCD in elderly patients undergoing total knee arthroplasty, and unveiling a possible mechanism by which parecoxib exerts anti-inflammatory and analgesic activities.

Accumulating evidence demonstrates a pivotal role of neuroinflammation in the POCD process. Proinflammatory cytokine release and astrocyte activation are associated with declined cognitive performance in humans and animals.^[5–7]^ Indeed, proinflammatory cytokines, such as TNF-α, IL-1β, and IL-6, can be released by activated astrocytes, triggering neuroinflammation and leading to cognitive dysfunction.^[9]^ High concentrations of proinflammatory cytokines inhibit long-term potentiation and impair memory.^[14]^ Neuroinflammation has been implicated in cognitive impairment; this may provide a viable target to prevent the development of POCD. Animal studies suggested COX-2 inhibitors to be promising candidates for the treatment of neuroinflammation and cognitive decline caused by surgical trauma.^[11,12]^ In the current study, the plasma levels of pro-inflammatory cytokines were significantly elevated after surgery in both parecoxib and control groups, but the degree of increase in the parecoxib group was markedly lower compared with that of controls. We speculate that the prophylactic effect of parecoxib on POCD likely results from its anti-inflammatory activity. However, more studies are needed to further understand parecoxib's effects.

Postoperative acute pain is another potential risk factor for cognitive dysfunction. Animal studies demonstrated that postoperative acute- or inflammatory pain could exacerbate memory deficits.^[9,10]^ Available evidence indicates that ketoprofen, a nonsteroidal anti-inflammatory drug (NSAID), can prevent the development of surgery-associated cognitive dysfunction via its pain-relieving effects in aged rats.^[15]^ Parecoxib sodium is frequently used for postoperative analgesia because of less side effects. In the current study, parecoxib decreased postoperative pain scores in patients after surgery. Furthermore, parecoxib reduced POCD incidence in aged patients. Therefore, the effectiveness of parecoxib in reducing POCD incidence might also be explained by its analgesic activity, which alleviates cognitive deficits.

There are several limitations of this study that should be mentioned. First, sample size was relatively small to fully demonstrate the effectiveness of parecoxib. No statistically significant difference was found in POCD incidence between both groups at 3-month follow-up, which may result from the reduced sample size. In addition, POCD is a common neurological complication of surgery; therefore, cerebrospinal fluid samples may be more suitable than plasma specimens for assessing proinflammatory cytokines levels. Furthermore, despite randomization, fewer smokers were found in the control group compared with the parecoxib group. It is unknown whether smoking would affect such studies, but it was recently shown that nicotine, the main specific alkaloid in tobacco smoke, does not alter cognitive function.^[16]^ Ongoing work in our laboratory aims at addressing these issues.

In conclusion, parecoxib decreased POCD incidence after total knee arthroplasty in elderly patients. Anti-inflammatory therapy and effective postoperative pain control with parecoxib may have benefits in preventing POCD in elderly patients. The current study provides a strong basis for parecoxib use as a prophylactic drug for POCD prevention.
