# Parecoxib prevents early postoperative cognitive dysfunction in elderly patients undergoing total knee arthroplasty: A double-blind, randomized clinical consort study: Erratum

**DOI:** 10.1097/MD.0000000000012065

**Published:** 2018-08-17

In the article, “Parecoxib prevents early postoperative cognitive dysfunction in elderly patients undergoing total knee arthroplasty: A double-blind, randomized clinical consort study”,^[1]^ which appeared in Volume 95, Issue 28 of *Medicine*, “9.7% vs 6.7%” should be replaced with “13.3% vs 8.5%” in the abstract section. Figure 1 and Table 3 were revised and should appear as:

**Figure 1:**
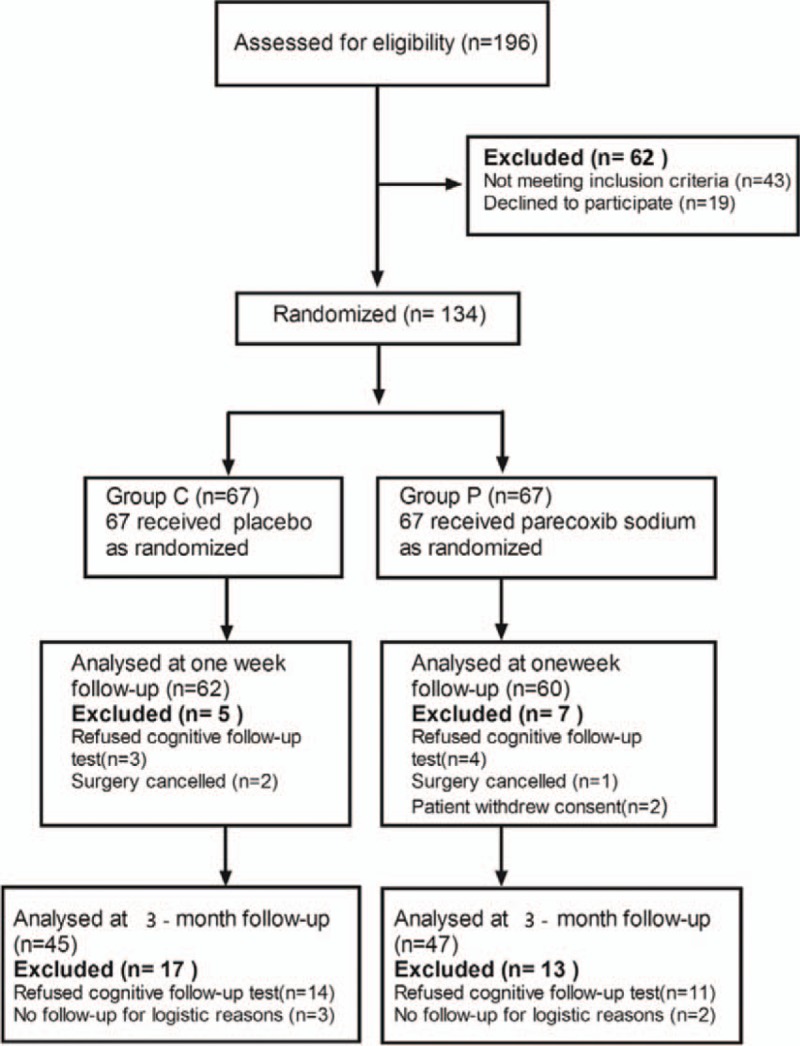
Consort diagram of patients’ randomization, intervention, and analysis.

**Table 3:**
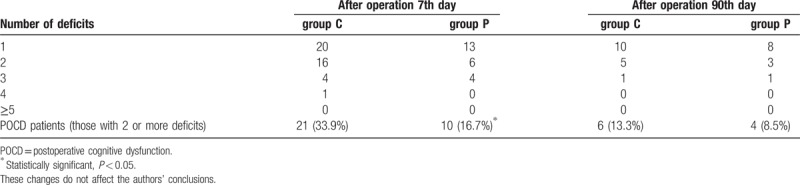
Patients with postoperative neuropsychological deficit in the test battery at baseline, 7 days, and 90 days follow-up.
